# Localized cystic kidney disease: a case report unveiling clinical and histopathological challenges

**DOI:** 10.4322/acr.2024.498

**Published:** 2024-06-21

**Authors:** Teresita Mendez, Hisham F. Bahmad, Francesca Polit, Nicole Carpio, Arman Gill, William F. Burke, Akshay Bhandari, Robert Poppiti, Yumna Omarzai

**Affiliations:** 1 Florida International University, Herbert Wertheim College of Medicine, Miami, FL, USA; 2 Mount Sinai Medical Center, Arkadi M. Rywlin M.D. Department of Pathology and Laboratory Medicine, Miami Beach, FL, USA; 3 Columbia University, Mount Sinai Medical Center, Division of Urology, Miami Beach, FL, USA; 4 Mount Sinai Medical Center, Department of Diagnostic Radiology, Miami Beach, FL, USA; 5 Florida International University, Herbert Wertheim College of Medicine, Department of Pathology, Miami, FL, USA

**Keywords:** Case Reports, Kidney Diseases, Polycystic Kidney Diseases, Polycystic Kidney, Autosomal Dominant, Kidney Diseases, Cystic

## Abstract

Localized cystic kidney disease (LCKD) is a distinct renal disorder characterized by the presence of cysts within specific regions of the kidneys. We present a rare case of a 41-year-old African American man, who presented to our medical center with lower urinary tract symptoms and gross hematuria. The initial assessment culminated in the identification of an uncomplicated urinary tract infection, prompting the prescription of appropriate oral antibiotic therapy. On follow-up after 5 months, the patient presented with gross hematuria. Imaging studies revealed a mixed-density cystic lesion of 2.6 cm situated within the interpolar region of the right kidney. This cystic lesion exhibited intricate septations at the superior pole of the kidney. Robotic-assisted right partial nephrectomy was performed, and pathologic examination was diagnostic for LCKD. This report not only underscores the uniqueness of LCKD but also presents a comprehensive review of the existing literature that pertains to this condition. Particular emphasis is placed upon its inherent benign behavior and its marked divergence from the progressive trajectory commonly associated with other renal diseases. We also explored the incidental findings of the disease, its diverse clinical symptomatology, conceivable etiological underpinnings, and the array of diagnostic modalities used. Finally, similarities in histopathologic findings with polycystic kidney disease and other entities are discussed, underscoring the importance of accurate diagnosis and management.

## INTRODUCTION

Localized cystic kidney disease (LCKD) represents a unique entity among renal cystic diseases. It is characterized by the development of cysts confined to specific regions within the kidney. The condition was first described in 1979 by Cho et al.,^1^ and has been referred to by many names, most commonly unilateral polycystic kidney disease and segmental polycystic kidney disease.^2,3^ However, the inclusion of the term “polycystic” in its name can be misleading and has the potential for a misdirected correlation with autosomal dominant polycystic kidney disease (ADPKD). Unlike more aggressive kidney disorders such as polycystic kidney disease, LCKD is an entity that refrains from inflicting renal degeneration or progressing to a decline in renal function.

We present the case of a 41-year-old African American man with Sickle Cell trait who was initially seen by Urology for an uncomplicated urinary tract infection and was later worked up for gross hematuria. He was found to have a discrete 2.6 cm mixed-density cystic lesion in the interpolar region of his right kidney. A robotic-assisted right partial nephrectomy was performed, and histopathological analysis confirmed the diagnosis of LCKD. This case report highlights the sporadic diagnosis of LCKD and offers a comprehensive review of the existing literature. This surgical case report was conducted and reported by Surgical CAse REports (SCARE) guidelines for case reports.

## CASE REPORT

A 41-year-old African American man, never smoker, with sickle cell trait and no other medical history, presented to the Emergency Department complaining of urinary frequency, dysuria, and gross hematuria. He denied fever, chills, or flank pain. On presentation, he was afebrile and hemodynamically stable. The physical exam was unremarkable.

Urinalysis revealed amber-colored urine positive for nitrite, leukocyte esterase, 38 white blood cells (WBCs) per high power field (HPF), WBC clumps, and budding yeasts, consistent with a urinary tract infection. PCR testing for chlamydia and gonorrhea were negative. The patient was sent home on empiric antibiotics, and the urine culture yielded *Escherichia coli* growth of >100,000 CFU/mL, for which he was properly treated.

**Figure 1:**
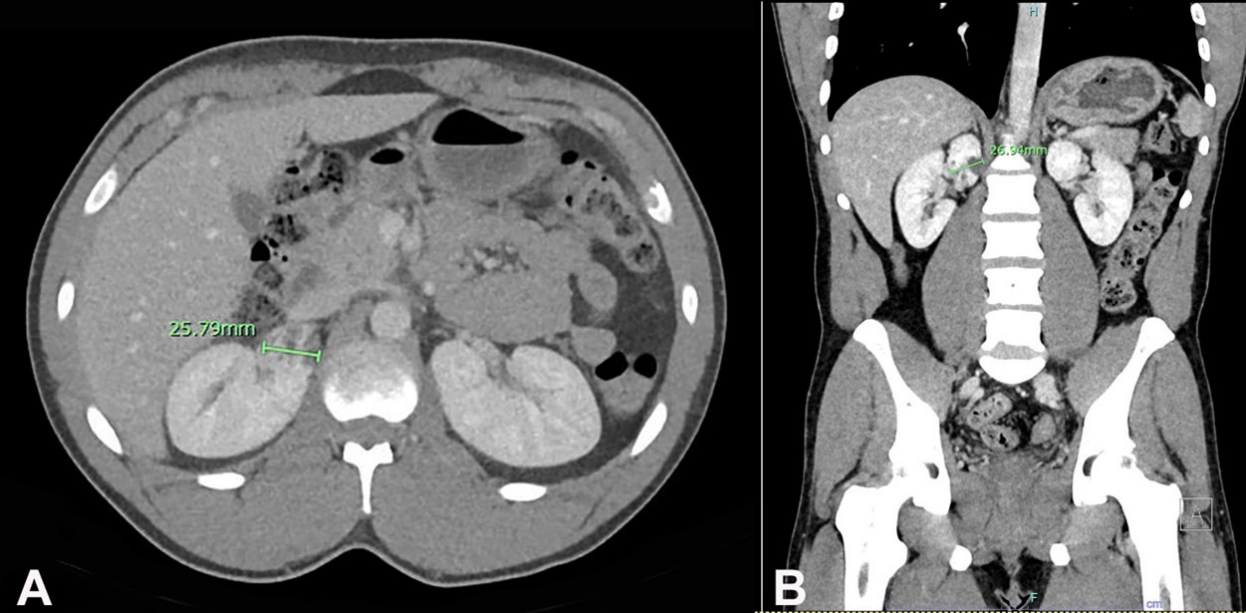
**A** and **B** – Computed tomography (CT) urography demonstrating a 2.6 cm mixed density lesion with heterogeneous enhancement of the upper pole of the right kidney.

On follow-up after 5 months, the patient presented with a chief complaint of gross hematuria despite negative urine workup and studies for sexually transmitted diseases (STD). The patient underwent computed tomography (CT) urography for further evaluation of his hematuria. The CT imaging demonstrated a 2.6 cm mixed-density lesion with heterogeneous enhancement of the upper pole of the right kidney (Figure 1).

**Figure 2:**
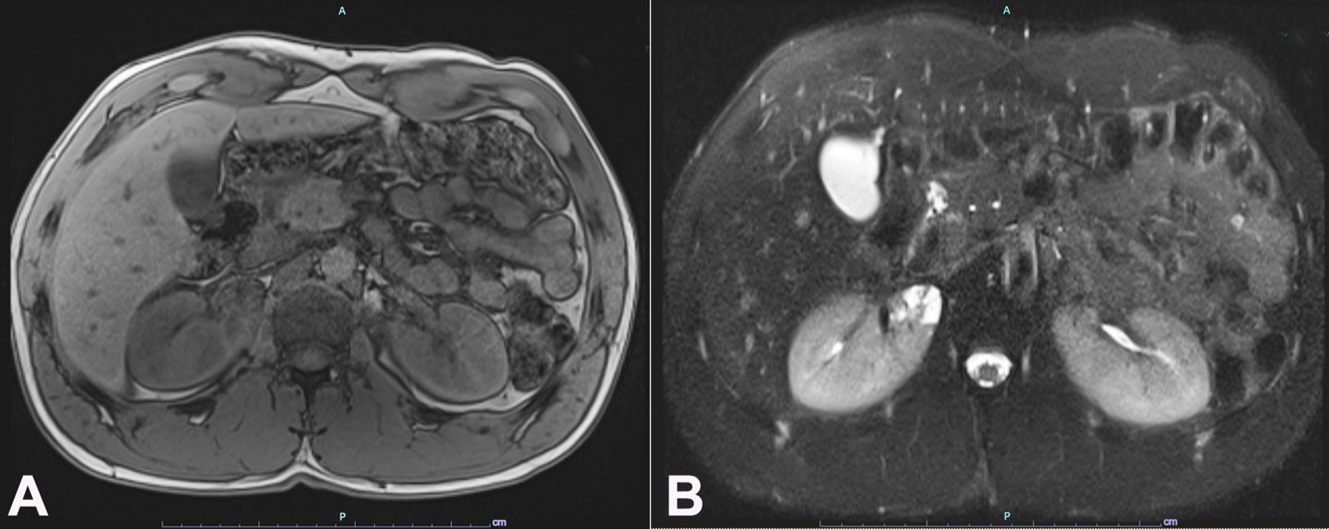
**A** and **B** – Magnetic resonance imaging (MRI) of the right kidney showing a 2.7 cm cystic lesion with thickened and enhancing septation (Bosniak III) (**A** – T1 weighted in-phase sequence, **B** – T2 weighted fat-suppressed sequence).

The differential diagnosis included malignancy, infection, and infarction. Magnetic resonance imaging (MRI) of the kidney confirmed the 2.7 cm right renal cystic lesion with thickened and enhancing septation (Bosniak III) (Figure 2).

The risks and benefits of active surveillance or intervention were discussed with the patient, but there was a concern for an atypical renal cell carcinoma such as medullary RCC,^4^ given the patient’s history of sickle cell trait, race, and age.^4^ The joint decision between Urology and the patient was to proceed with surgery.

**Figure 3:**
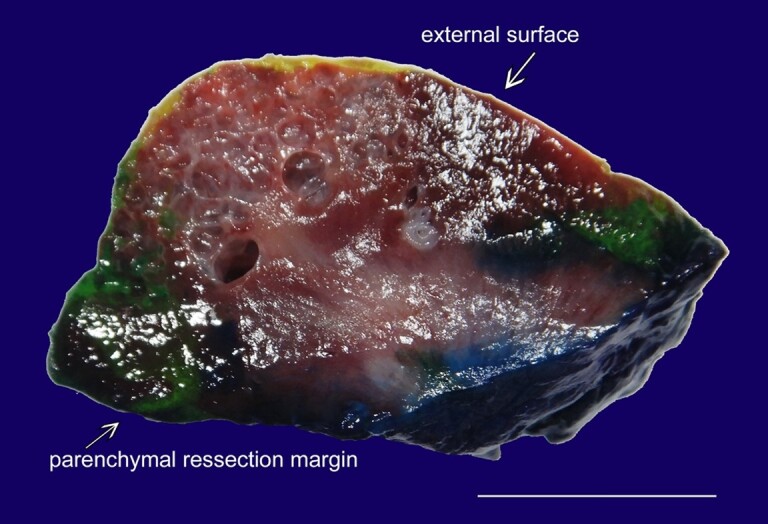
Gross image of the partial nephrectomy specimen. Cross section reveals a brown multi-loculated cystic mass (1.8×1.5×1.1 cm). The cysts have smooth and glistening walls and no excrescences (scale bar = 1 cm).

Robotic-assisted right partial nephrectomy was performed after preoperative intravenous (IV) Cefazolin antibiotic therapy. The patient tolerated the procedure well without any post-procedural complications. Gross pathologic examination showed a specimen that weighed 8 grams and measured 3.3×2.3×1.7cm. A mass was seen bulging from the outer surface of the specimen. Sectioning revealed a brown multi-loculated cystic mass that measured 1.8×1.5×1.1cm (Figure 3).

The cysts contained clear yellow fluid and had smooth and glistening walls without excrescences. Microscopically, hematoxylin and eosin (H&E)-stained sections demonstrated cysts of various sizes lined by flattened epithelium and adjacent normal renal parenchymal tissue separating the cysts. Immunohistochemical (IHC) stains in the extensively attenuated and bland epithelial lining cells of the renal cysts, excluding a vascular lesion, were positive for PAX8 while negative for D2-40, CD31, and CD34. In addition, IHC stains for ER and PR were negative and failed to reveal any definitive evidence of ovarian stroma in the intervening hyalinized tissue containing atrophic renal parenchyma between the cysts, excluding the diagnosis of a predominantly cystic mixed epithelial and multicystic stromal tumor (MEST)/adult cystic nephroma (CN).

**Figure 4:**
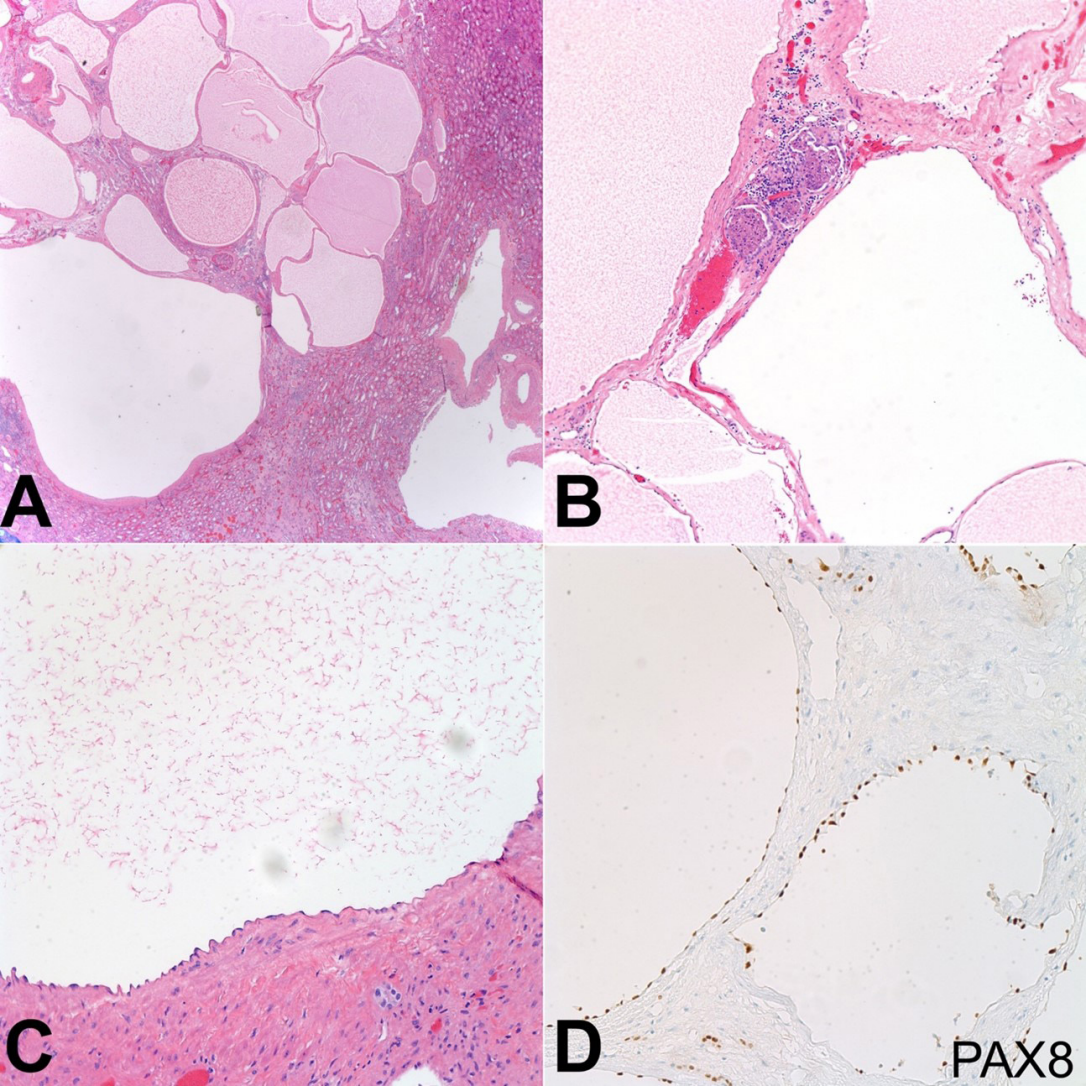
Microscopic images of the lesion. **A**, **B** and **C** – show hematoxylin and eosin (H&E)-stained sections of the lesion, which is composed of cysts of various sizes lined by flattened epithelium and an adjacent normal renal parenchymal tissue separating the cysts; **D** – shows immunohistochemical (IHC) stains that are positive for PAX8.

Together, the gross, histologic, and immunohistochemical findings yielded the diagnosis of LCKD (Figure 4). The patient was discharged and at 3-months follow up, he remained asymptomatic and demonstrated no signs of any complications or adverse effects.

## DISCUSSION

Localized cystic kidney disease (LCKD) is an uncommon entity that is identified incidentally during imaging studies performed for unrelated reasons. It has traditionally been observed in adults, with an average age at presentation in the mid-50s. However, there have been documented case reports of LCKD occurring in younger patient populations as well.^2,5^ In a study by Yoon et al.,^2^ 11 cases of pediatric LCKD were reported. These cases included infants as young as 6 months and 20 days of age with no family history of ADPKD. Importantly, the symptoms primarily affected the kidneys, distinguishing LCKD from systemic conditions like ADPKD, which can involve other organs. Regardless of age, the clinical symptoms range from asymptomatic to the presence of hematuria, flank pain, and palpable abdominal masses.^2,6-12^

The pathology and pathogenesis of LCKD remain unclear and offer an area of ongoing research and investigation. Several etiological factors have been proposed, including acquired factors, congenital abnormalities, and somatic mutations. Many studies highlight a male predominance in the cohorts studied. The pediatric reports of LCKD suggest a congenital component and possible maldevelopment of the kidney, leading to the development of this entity.^3,6^ Other reports propose a somatic mutation of *PKD1* (Polycystin 1, Transient Receptor Potential Channel Interacting), which is a gene responsible for the majority of cases of ADPKD, although LCKD differs in its disease progression.^12^ Nonetheless, the exact mechanisms underlying cyst formation within localized areas of the kidneys are yet to be fully elucidated. While the existing literature suggests potential causes, further studies are needed to establish definitive etiological factors.

Study reports consistently describe similar histologic findings after patients undergo nephrectomy for a suspected renal neoplasm. Pathologically, LCKD exhibits some common histologic findings with polycystic kidney disease.^11^ The multilocular cysts of LCKD are of variable sizes and are lined by flattened epithelial cells. They are surrounded by unremarkable renal parenchyma, and the cysts are filled with clear yellow fluid with no evidence of tumor formation.^2,11^ In the original study by Cho et al.,^1^ microscopic image demonstrated dilatation of ducts and tubules of the kidney forming cysts lined by flattened to cuboidal bland epithelium. No mitotic activity nor necrosis was seen. These microscopic similarities pose diagnostic challenges, emphasizing the need for careful histologic examination as the diagnosis is mainly made based on morphology on H&E with no major role in immunohistochemistry or other ancillary studies.

To assist in diagnosis and evaluation, a combination of imaging techniques is employed to assess LCKD. Ultrasound (US), CT, and MRI all play pivotal roles in facilitating accurate diagnosis and providing detailed information regarding the size, location, and composition of the cysts. These imaging modalities enable precise localization and differentiation of LCKD from other renal disorders.

Studies have demonstrated that despite being classified as “localized,” the cysts associated with this condition do not exhibit a specific predilection for a particular location within the kidney. Multiple reports show that multi-sized cysts can be found in various regions, including the upper pole, lower pole, and mid-portion of the kidney. The contralateral kidney is an internal control as it is usually unaffected. It is important to note that the kidney’s physiologic or age-related cystic change may confound evaluation. US findings typically show multiple, variably sized, round cysts with adjacent normal renal parenchyma.^2^ CT findings may also reveal focal calcifications within the cyst walls in both the pediatric and adult populations,^2^ which aids in differentiating it from other entities such as multilocular cystic nephroma (MLCN), which preferentially affects boys in the pediatric population and women in the adult population.^13^ Angiograms can also be utilized to assess LCKD, revealing avascular masses devoid of neovascularity. In addition, the angiograms typically show normal vasculature in the unaffected regions of the kidney.^11^

Radiologists need to become familiar with LCKD and its presentation on CT findings. The utilization of the Bosniak classification system can aid in appropriately categorizing risk-stratified cystic lesions.^14^ This classification system, which assigns grades I-V to the lesions on CT, MRI, and, to some extent, US, helps guide the selection of the most suitable therapeutic interventions based on the identified level of risk.^7,9,10,14^ With the appropriate classification, unnecessary surgical intervention may be avoided.

Localized cystic kidney disease is a distinct renal entity characterized by the presence of cysts within regions of the kidney. Compared to other kidney disorders, it typically follows a functionally nonprogressive course. The disease is often incidentally discovered, and its clinical presentation ranges from asymptomatic cases to specific renal manifestations. Although the precise etiology remains uncertain, potential acquired, congenital, or somatic mutation-related factors have been proposed. Pathological findings resemble those observed in polycystic kidney disease, highlighting the importance of accurate imaging techniques, particularly CT and MRI imaging, in diagnosing and managing LCKD.

## CONCLUSION

Further research is necessary to unravel the underlying mechanisms, establish definitive etiological factors, and optimize diagnostic and therapeutic approaches for LCKD. An awareness of and deeper understanding of this condition will contribute to a better understanding of its pathogenesis and, ultimately, improved patient outcomes, which may include kidney preservation in this benign condition.
